# Simple centrifugal fractionation to reduce the size distribution of cellulose nanofibers

**DOI:** 10.1038/s41598-020-68642-7

**Published:** 2020-07-16

**Authors:** Lindong Zhai, Hyun Chan Kim, Jung Woong Kim, Jaehwan Kim

**Affiliations:** Center for Nanocellulose Future Composites, Department of Mechanical Engineering, Inha University, 100 Inha-Ro, Michuhol-Ku, Incheon, 22212 Korea

**Keywords:** Engineering, Materials science, Nanoscience and technology

## Abstract

Since cellulose nanofiber (CNF) has unique characteristics in terms of renewability, high specific elastic modulus and strength and transparency, it is attractive for a building block of future materials. CNF can be extracted from various natural resource by several means. However, the size of the extracted CNF is very broad and uniformity of the extracted CNF is very important for many applications. Thus, a fractionation process is necessary to obtain a uniformly sized CNF. In this paper, a simple centrifugal fractionation was carried out to reduce the size distribution of the extracted CNF suspension from hardwood pulp by the combination of TEMPO oxidation and aqueous counter collision methods. The original CNF suspension was diluted and centrifuged under low speed to remove cellulose microfibers then centrifuged under high speed to separate very small CNF. The centrifugation condition is 10 k rpm for 1 h followed by 45 k rpm for 4 h. The fractionated CNF was analyzed by an atomic force microscopy, and the length and width distribution histogram analysis was utilized. UV–visible analysis, FT-IR and XRD crystallinity analysis were carried out to analyze all fractionated CNFs and the original CNF. After centrifugal fractionation, the width and length distribution range were reduced by 62% and 70%, respectively. It is shown that the centrifugal fractionation is an easy and efficient method to fractionate a uniform CNF suspension.

## Introduction

The tallest tree in the world which is 115 m high sustains itself for more than 600 years under its own weight and harsh environment of winds, snows, and rains^1^. Wood is a fiber reinforced polymer composite produced in nature, which exhibits marvelous structural behaviors. It is a natural composite of cellulose fibers embedded in a matrix of lignin and hemicellulose. Typically, cellulose fibers in the wood are hierarchically composed of macrofibrils and microfibrils surrounded by hemicellulose and lignin. The microfibril is formed with crystalline domain and short amorphous domain^2^. The microfibril, so called cellulose nanofiber (CNF), has unique characteristics in terms of renewability, high specific elastic modulus and tensile strength, low thermal expansion coefficient^3^. During the biosynthesis process, cellulose molecules can form into microfibrils through the formation of inter- and intra- molecular hydrogen bonds^4^. CNF is a material composed of nanosized cellulose fibrils with a high aspect ratio (length to width ratio). The width of CNF is typically in the range of 5–20 nm and length is typically up to microns^5,6^. CNF is attractive for many applications, for instance, coatings, fillers, additives, structural composites, cosmetics, flexible electronics and flexible displays^7–12^. CNF extracted from plants by top-down approach can be a building block of future materials. CNF can be extracted by mechanical, chemical and biological methods^13–16^. However, the length of the extracted CNF is very broad depending on the extraction method and pretreatment of cellulose resources. Uniformity and size controllability of the extracted CNF is very important for CNF applications. CNF can be utilized in films, filaments and substrates for flexible electronic devices. The uniformity of CNF affects not only the mechanical properties but also the surface roughness. Low surface roughness is an important requirement for polymer substrates for flexible OLED^17^. In the case of CNF filament applications if different sizes of CNFs are mixed together then it can cause defects in the filament, which result in lower mechanical properties^18^. Thus, an additional fractionation process over the CNF extraction method is essential. This process is called CNF fractionation. There are several fractionation methods, for example, field flow fractionation (FFF)^19,20^, gravitational sedimentation^21^ and centrifugal fractionation^22^. FFF is a fractionation method where a field is applied to a fluid suspension or solution pumped through a long and narrow channel, perpendicular to the direction of flow, to cause fractionation of the particles present in the fluid, depending on their differing ‘mobilities’ under the force exerted by the field^18^. In FFF, generally, the field can be asymmetric flow through a semi-permeable membrane, centrifugal force, thermal-gradient, electrical field and magnetic field. The asymmetric FFF is based on the size/molecular weight and it has only one semi-permeable membrane on the bottom wall of the channel. This cross flow is created by the carrier liquid exiting the bottom of the channel and offers an extremely gentle fractionation. Although this method can continuously fractionate, the fractionation range is broad.

This paper reports a simple centrifugal fractionation method that can efficiently reduce the size distribution of CNF. In the centrifugal fractionation method, centrifugal force acts as the field for the fractionation. This fractionation is based on the size and density of particles, which is a relatively easy and commonly used method in particle fractionation research. The centrifugal fractionation method has been used to fractionate rice straw cellulose nanofibrils produced by the aqueous counter collision (ACC)^23^ and cellulose nanocrystals (CNC) produced by an acid hydrolysis method^24^. This method can fractionate CNF in different sizes with simple equipment, but a continuous process is not easy. In this paper, CNF is extracted by using the combination of a TEMPO oxidation^25^ and ACC method and its size distribution are investigated. To improve its size uniformity, the centrifugal fractionation is utilized to fractionate a uniform CNF suspension by using a sequence of two centrifuge steps.

## Results and discussion

### Morphology observation

**Figure 1:**
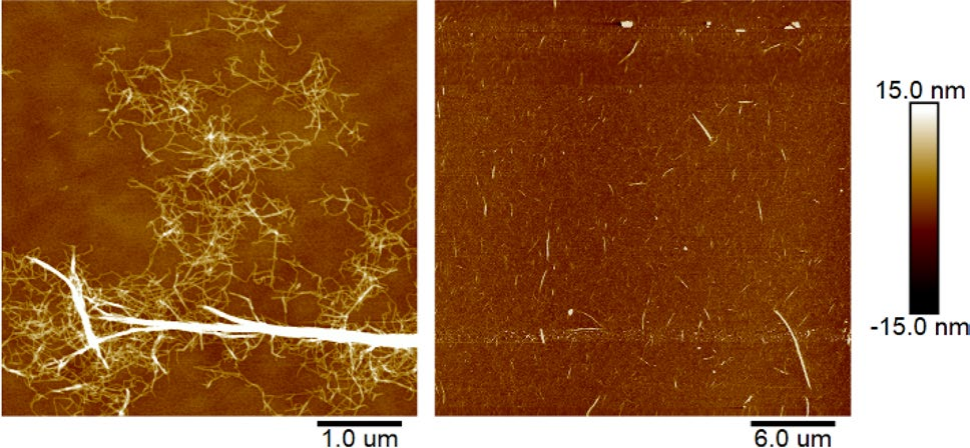
AFM images of original CNF suspension, 1 μm scale (left) and 6 μm scale (right).

CNF was successfully extracted by using the TEMPO oxidation and ACC combination method. The morphologies of the extracted CNFs were observed by using an AFM. Figure 1 shows the AFM images of the original CNF suspension with different scale bars. A high deviation in both length and width of the original CNF suspension can be observed. After the TEMPO oxidation and ACC combination process, most of extracted fibers were CNFs but still un-fibrillated fibers were remained in the suspension. Therefore, the centrifugal fractionation was carried out to eliminate the un-fibrillated fibers as well as very small sized ones.

**Figure 2:**
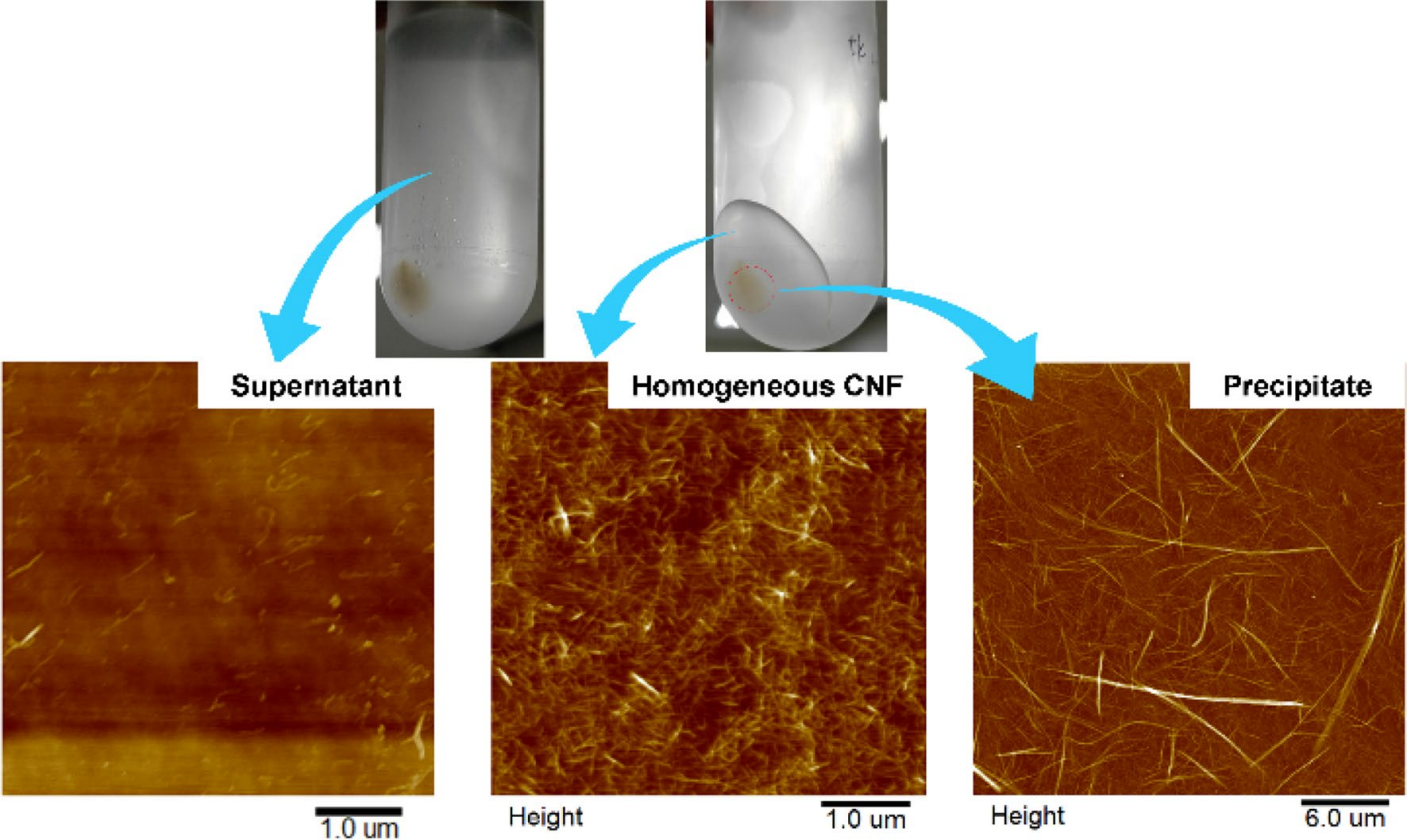
AFM images of fibers from each fraction after centrifugal fractionation, 0.1 wt% suspension of each fraction coated on cleaned silicon wafer.

After finishing the centrifugal fractionation, the supernatant was gently taking out by using a pipette, then the homogeneous CNF was separated subsequently, and finally, the precipitate remained in the centrifuge tube was collected. All fractions were investigated under AFM as shown in Fig. 2. As we expected, the supernatant, homogeneous CNF and precipitate were successfully separated. Small-sized CNF is the main part of supernatant and large-sized CNF is the main part of the precipitate. There are 6 centrifuge bottles for one-time centrifugal fractionation. Each centrifuge bottle contains 83 g of 0.5 wt% of the original CNF suspension. For calculating the yield of homogeneous CNF, the fractionated CNFs were separated from the centrifuge bottle and calculated the concentration to get the mass of the solid contents of each fraction. The yield of homogeneous CNF can be calculated by

1 $$Yield = \frac{Solid\;mass\;of\;homogeneous\;CNF}{{Solid\;mass\;of\;original\;CNF}} \times 100{\text{\% }}$$

The mass and concentration of homogeneous CNF were 5.6 g and 1.34 wt%, respectively. Therefore, the yield of homogeneous CNF was calculated to be 18.1%.

### Width and length distribution analysis

**Figure 3:**
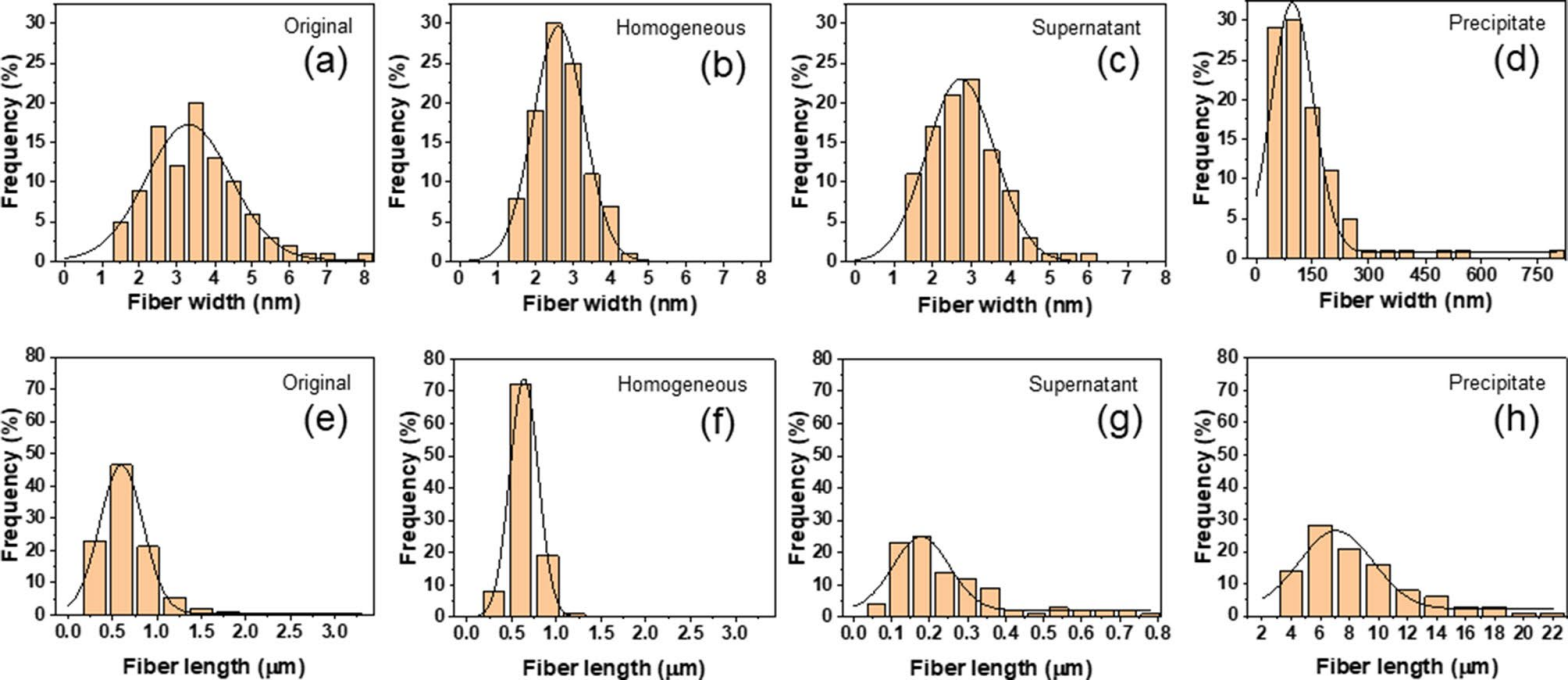
Width and length distribution histograms of the original CNF and all fractionated CNFs: (**a**) and (**e**) original CNF; (**b**) and (**f**) homogeneous CNF; (**c**) and (**g**) supernatant; (**d**) and (**h**) precipitate.

The length and width distribution histogram analysis was conducted to analyze all fractionated CNFs. The AFM images were taken into account for analyzing the width and length distributions of CNFs. Figure 3 shows the fiber width and length distribution histogram of all fractionated CNFs together with the original CNF. The width of the original CNF ranges from few nanometers to a few tens of nanometers and its length ranges from few tens of nanometers to micrometers. The average width and length of the original CNF are 3.5 ± 2.0 nm and 671.7 ± 961.2 nm, respectively. These results are similar to the previously reported results by Hai et al^6^. The variation ranges of the width and length of the original CNF are 6.6 nm and 3,000 nm, respectively. Such large deviations in length and width indicate that the original CNF might have a broad size distribution and there were un-fibrillated fibers as well as very small ones after the extraction. After the centrifugal fractionation, the width and length distributions of the homogeneous CNF were much narrower than the original CNF as displayed in Fig. 3a, b and e, f. The average width and length of the homogeneous CNF are 2.0 ± 0.6 nm and 639 ± 387.3 nm, respectively and the variation ranges of the width and length of the homogeneous CNF are 2.5 nm and 900 nm, respectively. This result indicates that the width and length distributions range were reduced by 62% and 70%, respectively. This kind of narrowing the size distribution is very important to prepare high quality CNFs. Note that the supernatant (see Fig. 3c, g) and precipitate (see Fig. 3d, h) were certainly fractionated. In summary, the centrifugal fractionation is an easy and efficient method to fractionate a homogeneous CNF suspension that has better uniform size distribution than the original CNF.

### Physical properties analysis

**Figure 4:**
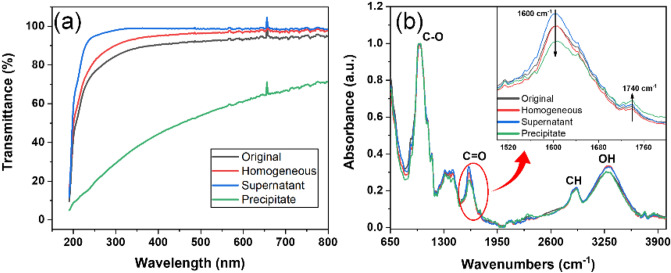
Transmittance and FT-IR analysis of all fractionated CNFs and original CNF.

UV–visible spectrophotometer, FT-IR and XRD analysis were carried out to investigate curves of all fractionated CNFs and the original CNF. Figure 4a shows the UV–vis results. As expected, in the visible light range (380–740 nm), the supernatant showed the highest transparency and the precipitate did the lowest transparency, which were 98.8% and 57.6%, respectively. Due to the presence of large-sized fibers in the original CNF, its transparency (92.8%) was similar but slightly lower than the homogeneous CNF (96.7%).

The FT-IR analysis was carried out for analyzing whether there is an effect on the chemical structure of CNF, the results are shown in Fig. 4b. The FT-IR analysis result was normalized according to C–O stretching band. The appearance of a C=O stretching band at 1,600 cm^−1^ and 1,740 cm^−1^ (carboxylate), C–O stretching band at 1,025 cm^−1^, CH stretching band at 2,900 cm^−1^ and a broad O–H stretching band at 3,400 cm^−1^ were observed in all fractions as well as in the original CNF. The peak related to carboxylate group was zoomed in as shown in Fig. 4b right corner. The peaks for all fractions and the original CNF were in the same position without shifting since they were from the same CNF suspension. Due to the prepared film thickness differences, the peak intensities were shown to be slightly different. Thus, the FT-IR analysis proves that the centrifugal fractionation did not affect the chemical structure of CNF.

Zeta potential is related to the surface charge density which is largely responsible for the colloidal stability of CNF in suspension. However, the zeta potential cannot be considered as a quantitative measure of surface charge density, but only as a relative assessment of colloidal stability. Generally, suspensions with absolute zeta potential values above 20 mV are considered colloidally stable. The measured Zeta potential values are − 52.81 mV (original CNF), − 52.56 mV (homogeneous CNF), − 40.45 mV (supernatant), and − 43.86 mV (precipitate), respectively. As we expected, the zeta potential of all fractions as well as original CNF are below − 40 mV, which indicate that they are colloidally stable. Moreover, the original CNF and homogeneous CNF suspension showed higher stability than that of supernatant and precipitate. This is due to large size distributions in supernatant and precipitate.

The carboxylate contents of each fraction as well as the original CNF were observed by using the conductometric titration method^26^ and Table 1 shows the results. As we expected, similar carboxylate content values were observed from all fractions and the original CNF. It might be due to all fractions were from the same suspension. The carboxylate content results are well corresponded with the FT-IR spectroscopy analysis results.

**Table 1:** Carboxylate content of all fractions as well as original CNF.

Sample	Carboxylate content (μeq g/g)
Original CNF	115.2
Homogeneous CNF	115.2
Supernatant	110.7
Precipitate	110.7

**Figure 5:**
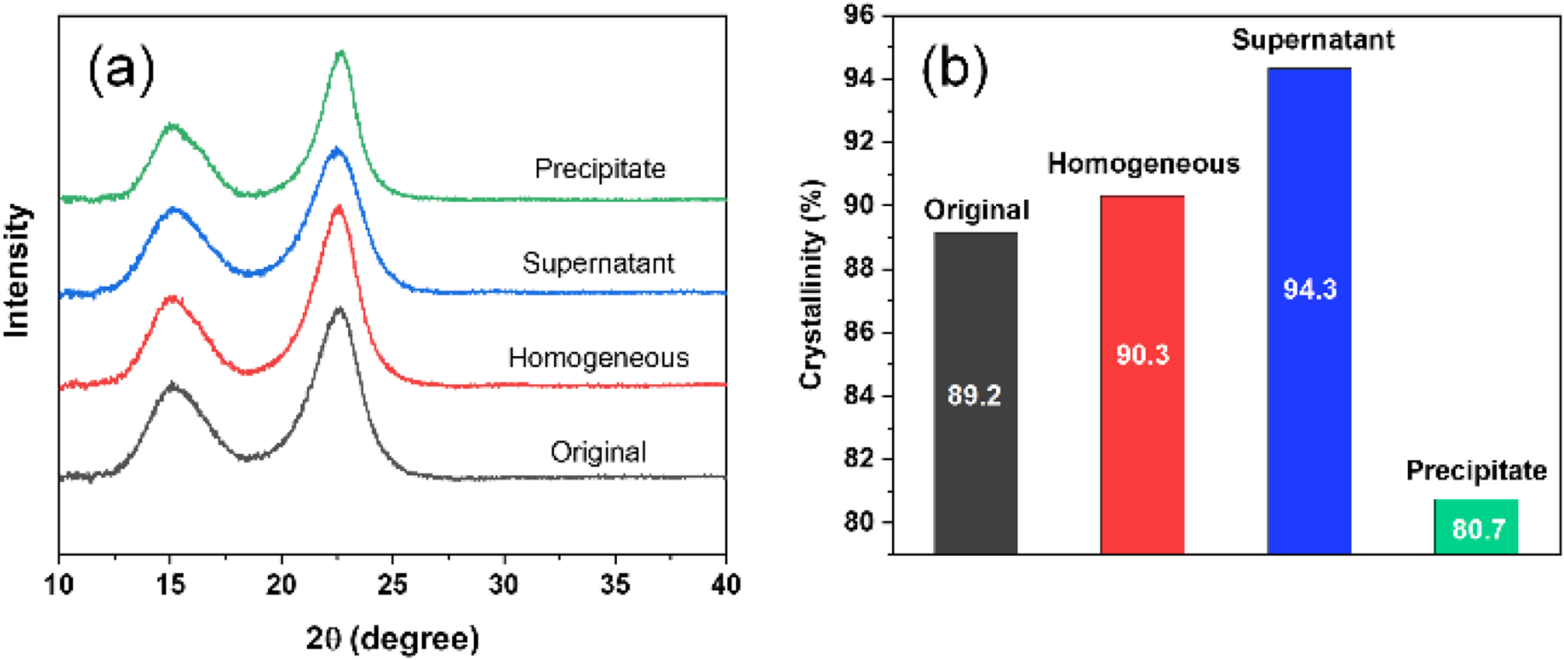
XRD pattern and crystallinity of all fractionated CNFs and original CNF.

The XRD analysis was conducted to observe the crystal structure differences of all fractionated CNFs and the original CNF. Figure 5a shows the results. All fractionated CNFs and the original CNF clearly show the 110 and 200 peaks. We can barely observe any differences in the XRD patterns. The calculated crystallinity values of all fractionated CNFs and the original CNF are shown in Fig. 5b. As expected, the supernatant shows the highest crystallinity (94.3%) and the precipitate shows the lowest crystallinity (80.7%), which is due to the fact that major contents of the supernatant are small-sized fibers and most of the large-sized fibers are contained the in precipitate. Generally, more amorphous cellulose parts are remained in the large-sized CNFs than the small-sized CNFs. Therefore, the precipitate exhibited the lowest crystallinity. After removing the precipitate, the crystallinity of the homogeneous CNF (90.3%) is slightly higher than the original CNF (89.2%).

## Materials and methods

### Materials

Bleached kraft hardwood (HW) pulp was received from Chungnam National University, South Korea (original source from Canada). It is in dried pad form and is a combination of Aspen and Poplar. 2,2,6,6-tetramethylpiperidin-1-oxyl (TEMPO), sodium bromide (NaBr), sodium hypochlorite (NaClO) and hydrochloric acid (HCl) were purchased from Sigma-Aldrich. Sodium hydroxide anhydrous (NaOH) was purchased from Daejung Chemical, South Korea.

### Methods

#### CNF extraction

CNF was extracted by taking the combination of the TEMPO oxidation method and the aqueous counter collision (ACC) method, which was reported previously^6^. In brief, HW pulp was swelled in deionized (DI) water for one day then disintegrated by mixer for 10 min. 5 g of cellulose, 0.0625 g of TEMPO, 0.625 g of NaBr and DI water were mixed together to get a 400 g suspension. The oxidation reaction started when 20 ml/g_cellulose_ of 15% NaClO was added into the mixture meanwhile stirred at room temperature. The pH was controlled at 12 by adding 0.5 M of NaOH solution. The pH value was measured by a pH meter (Orion star A211, Thermo Scientific). After 60 min, the oxidation reaction was stopped by adding 0.5 M HCl to adjust pH to 7. The oxidized cellulose then filtered by a filter paper with 0.45 um pore size to wash out the residue of chemicals. Finally, the oxidized cellulose was disintegrated by using ACC machine. This process acted as the pretreatment for the ACC treatment, which can reduce the initial size of raw cellulose fiber. The ACC treatment was taken by using the ACC machine (ACCNAC-100, CNNT, South Korea). 200 MPa water jets were used in the ACC machine and the TEMPO oxidized cellulose pulp was treated by the ACC system for 30 passes. The combination of the TEMPO oxidation and ACC method has advantages in terms of high extraction efficiency, low impact on the environment, low energy consumption and availability to mass production.

#### Centrifugal fractionation of CNF suspension

**Figure 6:**
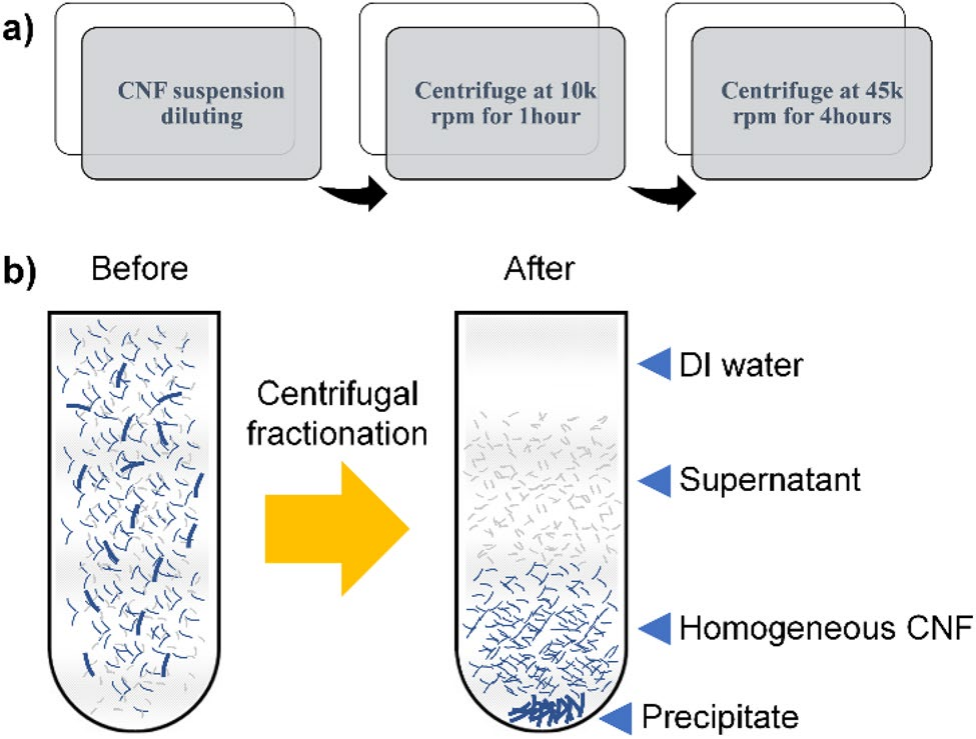
Centrifugal fractionation process: (**a**) flowchart and (**b**) schematic: after centrifugal fractionation, supernatant, homogeneous CNF and precipitate are fractionated.

2 wt% TEMPO 60 min oxidized, and ACC 30 pass treated CNF suspension was used for centrifugal fractionation. The TEMPO oxidation time and the number of the ACC pass were determined from our experience for the CNF extraction. The fractionation process is started by diluting the CNF suspension from 2 to 0.5 wt%. The centrifugal fractionation process is divided into two steps: 10 k rpm for 60 min and 45 k rpm for 4 h by utilizing an ultracentrifuge machine (CP 80NX, Hitachi, Japan). Figure 6 shows the flowchart and the schematic of the centrifugal fractionation process. The original CNF suspension has broad size distribution and after centrifugal fractionation, the CNFs are separated according to the size and a homogeneous CNF portion can be obtained. After centrifugal fractionation, as shown in Fig. 6b, the CNF suspension inside of the centrifuge tube can be separated into four layers: the DI water layer, small-sized CNF layer, homogeneous CNF layer and precipitate layer. Since the DI water layer and the small-sized CNF layer are mixed together, they cannot be separated, and it is called supernatant.

#### Characterization

The atomic force microscopy (AFM, Dimension3100, Bruker) was utilized to study morphology. Nanoscope and ImageJ software were conducted to characterize the length and width frequency distribution of all fractionated CNFs and original CNF. For the AFM morphology study, the original CNF suspension was diluted into 0.1 wt% and sprayed on a well cleaned silicon wafer by using a nitrogen gas spray gun. UV–visible spectrophotometer (8452A, HP) was utilized to characterize the transparency of all fractionated CNFs and original CNF. FT-IR spectroscopy analysis were recorded on an Agilent Cary 630 FTIR spectrometer to analyze the chemical structure before and after centrifugal fractionation. All fractions were fabricated in film forms for the FT-IR analysis. The FT-IR spectroscopy analysis is performed in typical wavenumber range 4,000–650 cm^−1^ with a 2 cm^−1^ resolution using 16 background scans and 16 sample scans under ATR mode. The refractive index of all fractions was set as 1.3328. For characterizing surface properties of all fractions, the zeta potential measurement was carried by using a Zeta potential analyzer (ELS-Z, Otsuka Electronics) at 0.1 wt% concentration at 25 °C and pH = 6.

The carboxylate contents of each fraction as well as the original CNF were observed by using the conductometric titration method^26^. In brief, each fraction and original CNF were prepared under the same condition (0.1 wt%, 150 mL) for the conductometric titration process. The pH of CNF suspension was adjusted to 2.5 with 0.01 M HCl under 10 min stirring to ensure the pH of suspension stable. This exchanges Na cations bound to the COOH group by H ions. The 0.01 M NaOH solution was titrated by using a micropipette (0.2 mL/min) into the suspension until the pH value reached to 11. The conductivity was monitored with a benchtop meter. The titration curve shows typical presence of strong and weak acid groups, and the amount of strong acid corresponds to the added HCl meanwhile the weak acid corresponds to the carboxylate content. The carboxylate content (CC) or degree of oxidation (DO) is given by the following equation:

2 $$DO\left( {CC} \right) = \frac{{162\left( {V_{2} - V_{1} } \right)c}}{{w - 36\left( {V_{2} - V_{1} } \right)c}}$$

where V 1 and V 2 are the equivalent volumes of the added NaOH solution (in L), c is the NaOH concentration (in mol/L), and w is the weight of dried sample (in g). The parameter of 162 corresponds to molecular weight of a glucose structural unit, and 36 is the difference between the molecular weight of anhydroglucose unit and that of sodium salt of a glucuronic acid moiety. A benchtop meter (Orion 4-star, Thermo Scientific) was utilized to monitor the conductivity.

X-ray diffractometer (XRD, X’Pert^3^ Powder, Malvern Panalytical) was carried out the measure the crystallinity of all CNFs. CuKα radiation source at 60 kV and 60 mA was selected. The crystallinity was calculated to analyze all fractionated CNFs and the original CNF. The X-ray diffraction deconvolution method (curve fitting) was utilized to calculate the crystallinity^27^. This method is based on the convolution of three sharp peaks, from $1\overline{1}0$, 110 and 200 crystalline diffractions and one broad peak representing the non-crystalline diffraction. Gaussian function is used for the deconvolution of the X-ray diffraction spectra. The crystallinity is found from the ratio of the area of all crystalline peaks to the total area including non-crystalline fraction following the equation:

3 $$Cr = \frac{{I_{cr peak1} + I_{cr peak 2} + I_{cr peak 4} }}{{I_{cr peak1} + I_{cr peak 2} + I_{{non{ - }cr peak}} + I_{cr peak 4} }} \times 100\%$$

where Cr is crystallinity, $I_{cr peak 1}$ and $I_{cr peak 2}$ are the areas under the first and second crystalline peaks corresponding to Miller index $1\overline{1}0$ and 110, respectively. $I_{cr peak 4}$ is the area under the fourth crystalline peak corresponding to Miller index 200 and $I_{non{\text{-}}cr peak}$ is the area under the non-crystalline peak. The peak deconvolution graphs are shown in Supporting Information (Fig. S1).

## Conclusion

HW CNF extracted by the TEMPO oxidation and ACC combination method was successfully fractionated by the centrifugal fractionation method. By analyzing the fractionation results, the centrifugation conditions (10 k rpm for 1 h and 45 k rpm for 4 h) were appropriate to fractionate CNF. The width and length distribution histograms were carried out to analyze the width and length distribution of the original CNF and all fractionated CNFs. The width and length distribution range of the original CNF were 6.6 nm and 3,000 nm, respectively. After centrifugal fractionation, the ranges of width and length of the homogeneous CNF were reduced to 2.5 nm and 900 nm, respectively, which correspond to 62% and 70% reduction, respectively. This kind of narrowing the size distribution is very important to prepare high quality CNF. The yield of the homogeneous CNF fraction was 18.1%. Furthermore, transparency and crystallinity analysis again confirmed the successful centrifugal fractionation. FT-IR spectroscopy analysis and carboxylate content analysis results indicated that the centrifugal fractionation method did not change the CNF itself and only separated the homogeneous CNF. This study indicates that the centrifugal fractionation is an easy and efficient method to fractionate a homogeneous CNF suspension.

This study aimed at to present a simple centrifugal fractionation method that can efficiently reduce the size distribution of CNF. This method can contribute to an industrial mass production of uniform CNF for further commercialization. The uniform CNF will be beneficial to produce ultra-strong and environmental-friendly fiber, which is not only essential for strong and tough natural composites but also for the field of biomedical applications, flexible electronics and energy devices.

## Supplementary information

Supplementary information.
