# Supplementary material for: Simple centrifugal fractionation to reduce the size distribution of cellulose nanofibers
Source: Sci Rep. 2020 Jul 16;10:11744. doi: 10.1038/s41598-020-68642-7 (PMC7366905; doi:10.1038/s41598-020-68642-7)
Supplement: Supplementary file 1 — Supplementary information. [file 41598_2020_68642_MOESM1_ESM.docx]

Simple centrifugal fractionation to reduce the size distribution of cellulose nanofibers

*Lindong Zhai^1^, Hyun Chan Kim^1^, Jung Woong Kim^1^, Jaehwan Kim^1*^*

^1^Center for Nanocellulose Future Composites, Department of Mechanical Engineering, Inha University, 100 Inha-Ro, Michuhol-Ku, Incheon 22212, Korea

SUPPLEMENTARY INFORMATION


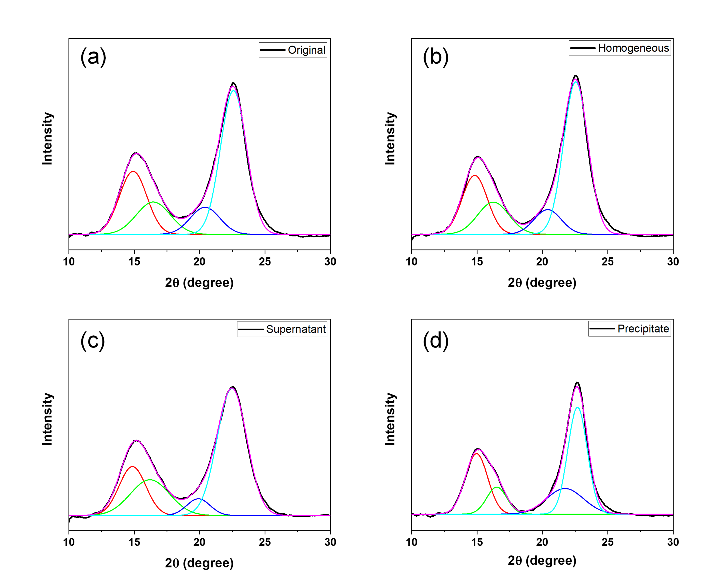


Figure S1. Peak deconvolution of the X-ray diffraction spectra. (a) original CNF, (b) homogeneous CNF, (c) supernatant, (d) precipitate.
